# A comprehensive analysis of immune features and construction of an immune gene diagnostic model for sepsis

**DOI:** 10.1186/s12864-023-09896-z

**Published:** 2023-12-20

**Authors:** Haiyan Xue, Ziyan Xiao, Xiujuan Zhao, Shu Li, Zhenzhou Wang, Jie Zhao, Fengxue Zhu

**Affiliations:** 1 Department of Critical Care Medicine, Peking University People’s Hospital, No. 11 Xizhimen South Street, Beijing, 100044 China; 2 National Center for Trauma Medicine of China, Beijing, China

**Keywords:** Sepsis, Infection, Immune disorder, Immune subtype, Diagnostic model

## Abstract

Sepsis is a life-threatening syndrome resulting from immune system dysfunction that is caused by infection. It is of great importance to analyze the immune characteristics of sepsis, identify the key immune system related genes, and construct diagnostic models for sepsis. In this study, the sepsis transcriptome and expression profiling data were merged into an integrated dataset containing 277 sepsis samples and 117 non-sepsis control samples. Single-sample gene set enrichment analysis (ssGSEA) was used to assess the immune cell infiltration. Two sepsis immune subtypes were identified based on the 22 differential immune cells between the sepsis and the healthy control groups. Weighted gene co-expression network analysis (WCGNA) was used to identify the key module genes. Then, 36 differentially expressed immune-related genes were identified, based on which a robust diagnostic model was constructed with 11 diagnostic genes. The expression of 11 diagnostic genes was finally assessed in the training and validation datasets respectively. In this study, we provide comprehensive insight into the immune features of sepsis and establish a robust diagnostic model for sepsis. These findings may provide new strategies for the early diagnosis of sepsis in the future.

**Supplementary Information:**

The online version contains supplementary material available at 10.1186/s12864-023-09896-z.

## Introduction

Sepsis is a type of multiple organ dysfunction syndrome (MODS) resulting from an imbalanced response to a severe infection [1]. Despite advancements and enhancements in critical care, sepsis remains the leading cause of morbidity and mortality among patients in intensive care units [2]. For instance, data revealed that there were approximately 48.9 million new diagnoses and 11.0 million sepsis-related fatalities in 2017 [2]. The risk of mortality escalates with the increase in time before initiating treatment, underscoring the critical importance of early diagnosis and effective therapy in improving patient outcomes [3]. Furthermore, biomarkers play a crucial role in diagnosing sepsis, facilitating early detection of organ dysfunction, identifying specific host response subgroups, planning appropriate therapy, and establishing prognoses [4, 5]. Thus, continued research into sepsis biomarkers and the development of biomarker-based diagnostic models are of paramount importance.

The pathogenesis of sepsis and the body's immune response are closely related. Nonetheless, this relationship is extremely complex. Previous research has indicated that sepsis typically involves the activation of the innate immune system, which includes factors such as the tumor necrosis factor (TNF-α), interleukin-1β (IL-1β), IL-6, IL-8, and interferon-γ (IFN-γ), as well as the acquired immune system, which manifests in the form of apoptosis of immune cells, specifically dendritic cells (DCs), natural killer cells, lymphocytes, neutrophils and antigen-presenting cells (APC) [6–8]. The onset and progression of sepsis are influenced by an imbalance in immune activation and immunosuppression. However, a comprehensive understanding of the molecular and cellular mechanisms responsible for sepsis-induced systemic immune dysregulation is still lacking. Recent advances in bioinformatics have led to increased exploration of the immunological landscape and the identification of immune-related biomarkers that may aid in early sepsis detection.

In this study, we aimed to enhance our understanding of the immunological features of sepsis and investigate the immunoregulatory mechanisms involved. To achieve this, we analysed the gene expression profiles of sepsis patients in the Gene Expression Omnibus (GEO) database and evaluated the status of immune cell infiltration. Using an adapted Lasso-Penalized Regression approach, we identified a set of 11 immune-related diagnostic genes. Subsequently, we constructed a diagnostic model based on these markers. To assess the predictive efficacy of the model, we then validated it using an independent cohort. Furthermore, we explored the relationship between the identified diagnosis-related genes and the infiltration of immune-related cells. This analysis provided valuable insights into the relationship between gene expression patterns and immune cell responses in sepsis patients.

Overall, our study elucidated the immunological characteristics of sepsis, paving the way for further investigations into the regulatory mechanisms underlying this complex condition. These findings provide a deep and significant understanding of the immune factors involved in sepsis, thus opening up new avenues for the development of novel strategies in early sepsis diagnosis.

## Materials and methods

### Data acquisition and processing

We used the GEO database (https://www.ncbi.nlm.nih.gov/geo/) [9] to extract datasets relevant to sepsis. After initial screening, GSE28750, GSE54514, GSE69063, and GSE69528 were selected for the training set, while GSE154918 was chosen for the validation set. Samples that met the criteria for either sepsis or normal controls were included in the study, while those that did not meet either criterion were excluded. For the included samples, the expression profiles were extracted for subsequent analysis. After extracting the expression profiles for the training set, we applied the combat function in the SVA package to integrate them, allowing for the elimination of batch effects [10]. Table 1 presents the basic information about these microarray datasets. The GSM accession numbers and grouping of the included samples are listed in Supplementary material 1.

**Table 1:** The characteristics of the datasets used in the present study

Dataset ID	Sepsis	Normal	Others	Platform
GSE28750	10	20	11	GPL570 [HG-U133_Plus_2] Affymetrix Human Genome U133 Plus 2.0 Array
GSE54514	127	36	0	GPL6947 Illumina HumanHT-12 V3.0 expression beadchip
GSE69063	57	33	63	GPL19983 [HuGene-2_1-st] Affymetrix Human Gene 2.1 ST Array [HuGene21st_Hs_ENTREZG_19.0.0]
GSE69528	83	28	27	GPL10558 Illumina HumanHT-12 V4.0 expression beadchip
GSE154918	24	40	41	GPL20301Illumina HiSeq 4000 (Homo sapiens)
**total**	301	157	142	

### ssGSEA and CIBERSORT

On a metagene set of 28 immune cells, single-sample gene set enrichment analysis (ssGSEA) was performed using the GSVA package [11]. The “immunological score” was calculated as a quantitative measure to demonstrate the enrichment level of metagenes in each sample, reflecting the intensity of infiltration of 28 immune cell types that correspond to the metagenes in the sample. The two-tailed Wilcoxon rank sum test was also carried out to examine the immunological scores and to differentiate between the 28 immune cell types in the two groups (*p* value 0.05). Using the normalized gene expression matrix of the sepsis samples, the deconvolution approach for cell-type identification by estimating relative subsets of RNA transcripts (CIBERSORT) was used to estimate the abundance of various immune cell types in each sepsis specimen [12].

### Unsupervised consensus cluster analysis

Consensus clustering was carried out using the "ConsensusClusterPlus" package and the process involved the enrichment analysis of the differential immune cells found in each sepsis sample. To perform the bootstrapping procedure, Pam arithmetic and the "Pearson" distance were used [13]. The optimal k was determined using the cumulative distribution function (CDF) and area under the receiver operating characteristic (ROC) curve (AUC) for the cluster number k, which ranged from 2 to 6. Subsequently, a chi-square test was performed in order to assess the survival proportions of the immunological subtypes, and the statistical significance was set to p 0.05. Additionally, the ssGSEA was employed to assess the results of the different paths utilizing GSVA package. Pathway activation was indicated by a mean normalized enrichment score (NES) > 0, and pathway inhibition was shown by NES < 0.

### Identification of Differently Expressed Genes (DEGs)

The Limma R package (with a threshold of |log2 fold change (FC)|> 1) was used to identify DEGs between the two immune subtypes and an adjusted *p* value < 0.05 for the selection of DEGs was established [14]. Moreover, the same package with a slightly different threshold |log2 fold change (FC)|> 0.263 and an adjusted *p* value of < 0.05 for differentially expressed gene selection was used to identify the DEGs between the sepsis samples and normal samples. The Benjamini–Hochberg method was employed to adjust the *p* values for multiple tests.

### Weighted Gene Co-expression Network Analysis (WGCNA)

The association between gene networks and diseases, as well as the identification of co-expressed gene modules among the DEGs, was investigated using the WGCNA method implemented via the "WGCNA" package [15]. To establish a scale-free distribution network, the "pickSoftThreshold" function in the WGCNA package was employed, allowing the determination of suitable soft powers within the range of 1–20. To reveal the connectivity between gene modules, the adjacency matrix was then converted into a topological overlap matrix (TOM). Additionally, hierarchical clustering was conducted, while different gene modules were represented in the form of coloured branches. The significance of the relationships between gene expression levels and different modules was calculated using the “minModuleSize” of 50 and “mergeCutHeight” of 0.3. Finally, the most significant modules were determined, and the characteristic genes within these modules were extracted for further analysis.

### Identification of Immune-related Differentially Expressed Genes (IRDEGs)

A total of 1811 immune-related genes (IRGs) were extracted from the Immunology Database and Analysis Portal (IMMPORT) database(https://www.immport.org/home). Meanwhile, the IRDEGs were identified by the overlapping genes among the IRGs, DEGs within the immunological subtypes, and the distinctive genes of the essential modules. These results were visualized and presented using a Venn diagram that illustrates the shared genes among these categories.

### Functional enrichment analysis

The DAVID (v.6.8) online database (https://david.ncifcrf.gov/summary.jsp) was employed to perform enrichment analyses based on Gene Ontology (GO) and Kyoto Encyclopedia of Genes and Genomes (KEGG) pathways [16–20]. The findings of the GO enrichment analysis were assigned to three categories (namely BP, biological process; CC, cellular component; and MF, molecular function). Finally, after a *p* value < 0.05 was selected for the threshold, the 10 most significant enrichment results were presented using histograms.

### Protein–Protein Interaction Network (PPI) Analysis

The STRING (v.10.0) online database (https://cn.string-db.org/cgi/) was employed to predict the relationships between the genes and PPI networks [21]. Subsequently, to further process the network diagram obtained via the STRING online database, Cytoscape (v.3.8.2) was applied.

### Construction of the diagnostic model

Univariable analysis and multivariable analysis were conducted to assess the association between the expression of each risk gene and the diagnosis of sepsis. This evaluation aimed to determine the prognostic value of these regulators in sepsis. The least absolute shrinkage and selection operator (LASSO) algorithm was used to confirm these risk genes, and the k-fold cross-validation approach was applied to identify the optimal penalty parameter. The algorithm below was used to calculate a risk score based on these three genes.

$$Risk\,Score=\sum (Coef(i)\times x(i))$$

In this equation, Coef(i) denotes the coefficient, while x(i) represents the relative expression value for the risk gene. Finally, to confirm that the model prediction process was accurate, an ROC curve was created. The R package “survivalROC” was applied to determine a value for the area under the curve (AUC).

### Expression validation and immunological correlation analysis

The expression values were obtained for both the training and validation datasets, after which a box diagram and heatmap were generated to visualize the discriminative effect. Additionally, the strength of the relationship between diagnostic genes and immune cells was determined using Spearman's rank correlation coefficient.

### Statistical analysis and workflow

**Fig. 1:**
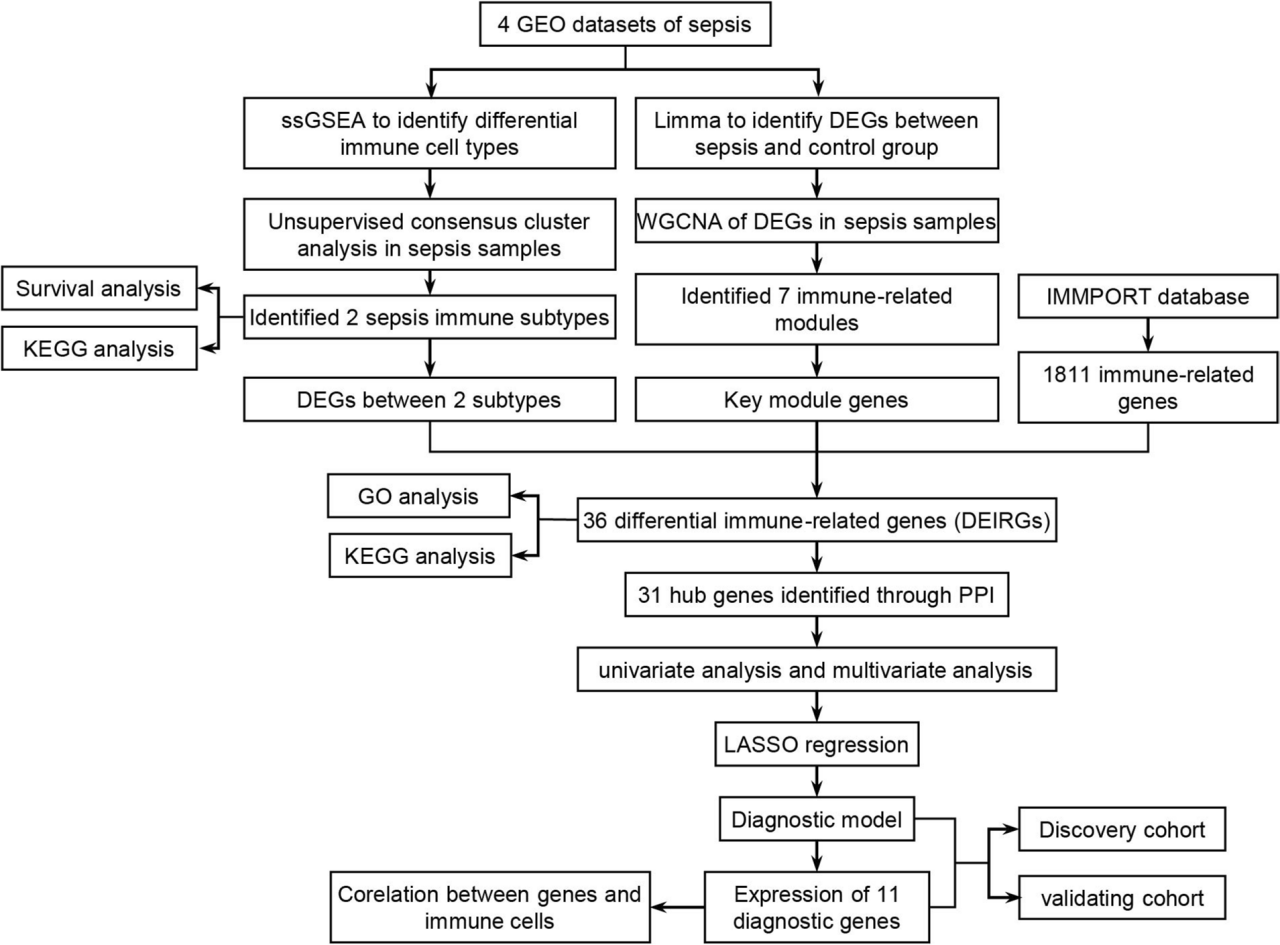
Workflow of this study

All the statistical tests carried out in this work were performed using R 3.6.1 (unless otherwise stated). Moreover, a *p* value < 0.05 was considered statistically significant (*p < 0.05, **p < 0.01, and ***p < 0.001). Figure 1 presents the overall workflow of this study.

## Results

### Preprocessing of data and the identification of differentially abundant immune cells

**Fig. 2:**
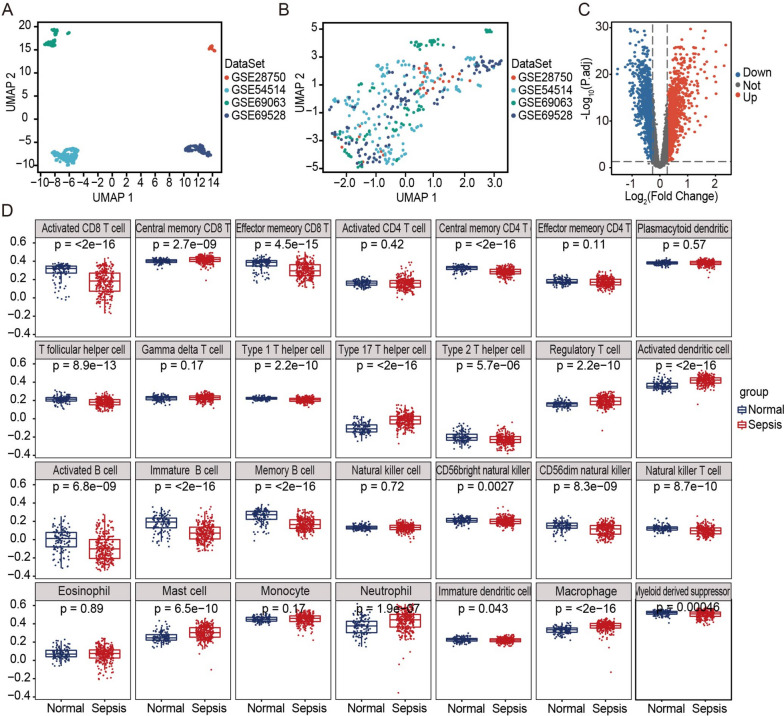
Analysis of the differentially abundant immune cell types within the integrated dataset. **A** Principal component analysis (PCA) before batch effect adjustment. **B** PCA after batch effect adjustment. **C** Volcano plot of DEGs between the sepsis and normal groups (*p* < 0.05). **D** Box plots displaying the relative proportions of 28 immune cell types in normal and sepsis patients

First, 277 sepsis samples and 117 normal control samples were extracted as the training set from the following four datasets: GSE28750, GSE54514, GSE69063, and GSE69528. Additionally, 24 sepsis samples and 40 normal control samples were extracted as the validation set from the GSE154918 dataset. Furthermore, 142 samples were excluded because they did not fit into either the sepsis or healthy group. For the training set, the SVA package was employed to eliminate the inter-batch differences and the expression matrix after batch effect correction was listed in Supplementary material 2. As shown in Fig. 2A, the batch effect had a significant impact on the clustering of samples. After applying SVA, the batch effect was mitigated (Fig. 2B). Altogether, 2484 DEGs were identified, including 1214 upregulated genes and 1270 downregulated genes (adjusted *p* value < 0.05 and |log2 FC|≥ 0.263, Fig. 2C)—Supplementary material 3.

To investigate the connection between sepsis and the infiltration of immune cells, ssGSEA was conducted to evaluate 28 immune cell types. The distribution and proportion of different immune cells between the sepsis and normal control groups are shown in Fig. 2D. The abundance 22 immune cells was significantly different between the two groups, among which macrophages, neutrophils, mast cells, T helper 17 (Th17) cells, and regulatory T (Treg) cells showed significantly more abundance in sepsis samples (*p* < 0.001).

### Construction of immune subtypes in Sepsis

**Fig. 3:**
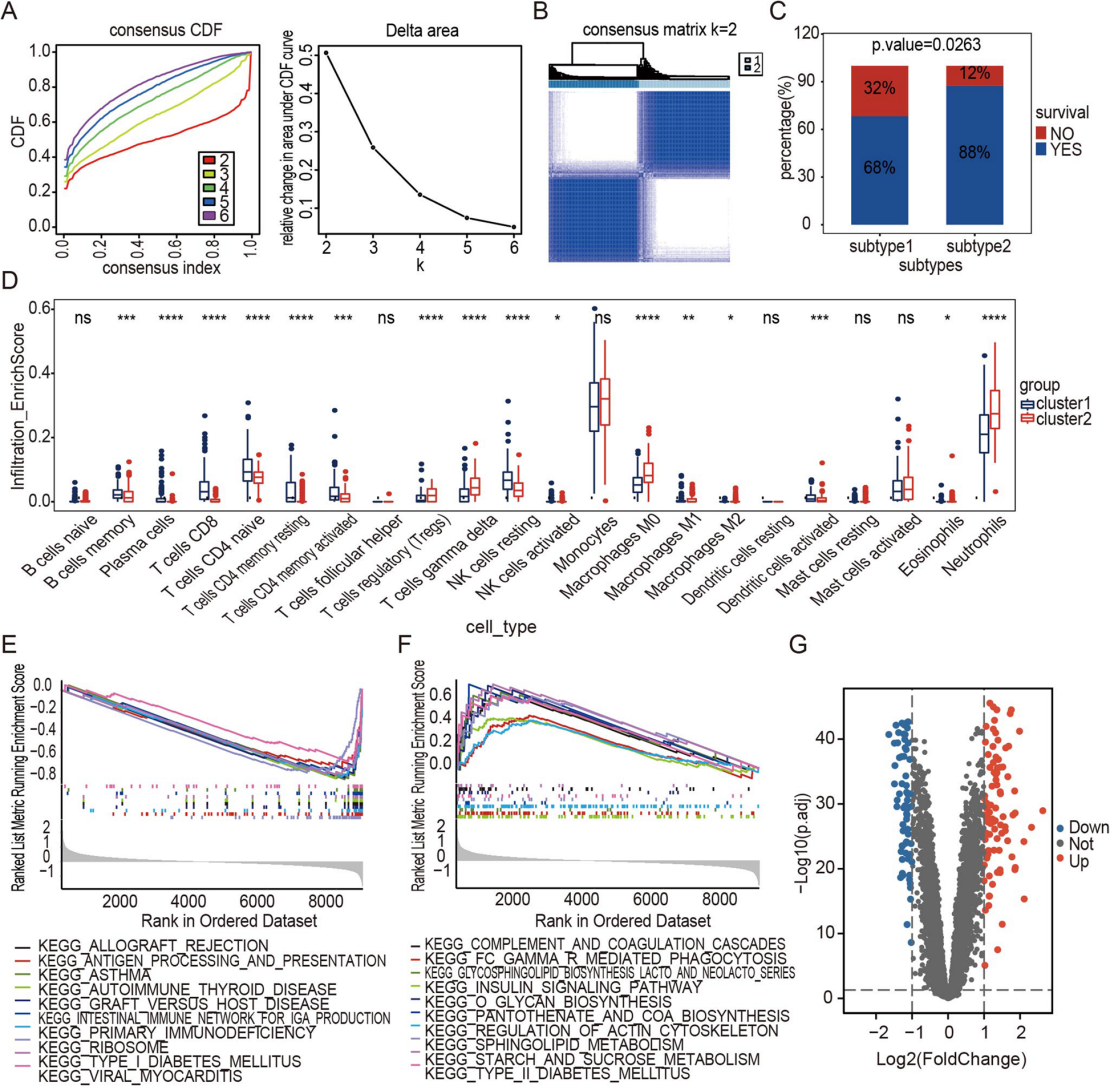
Consensus clustering of gene expression profiles of sepsis samples. **A** Cumulative distribution curves for subtypes with cluster counts (k) ranging from 2 to 6 and relative changes in the area under the CDF curve for each subtype. **B** The consensus matrix heatmap showing that the sepsis samples were classified into two subtypes. **C** Survival proportion analysis between the two subtypes (*p* = 0.0263). **D** Immune cell infiltration abundance in different subtypes. **E** The 10 activation pathways with the highest enrichment of upregulated DEGs (*p* < 0.05). **F** The 10 inhibition pathways with the highest enrichment of downregulated DEGs (*p* < 0.05). **G** Identification of the DEGs between the two subtypes (*p* < 0.05). The Wilcoxon rank sum test was used to compare the immune cells of the two subtypes. *p < 0.05, **p < 0.01, ***p < 0.001

Utilizing 277 sepsis samples, an unsupervised consensus cluster analysis was performed. The results (Fig. 3A, B) showed a high concordance of gene expression patterns in each cluster after 277 patients were divided into two subtypes. The consensus matrix heatmap is shown in Fig. 3B. Clinical characteristic analysis showed that the survival proportion in subtype 2 was higher than that in subtype 1 (Fig. 3C, *p* = 0.0263). The abundance of 22 immune cell populations was assessed using the CIBERSORT algorithm, showing that the abundance of neutrophils, M0 macrophages, M1 macrophages, and Treg cells was much higher in subtype 2 than in subtype 1 (Fig. 3D, *p* < 0.01). However, CD4 + T cells and CD8 + T cells were more abundant in subtype 1 than in subtype 2 (Fig. 3D, *p* < 0.01).

The biological role of immune subtypes was then investigated by examining the enriched pathways linked to them. The top 10 GSEA-enriched pathways were identified with a screening threshold of an adjusted *p* value of 0.05. (Figs. 3E and F). Moreover, the DEGs between the two subtypes were determined using standard threshold values of |log2 fold change (FC)|> 1 and an adjusted *p* value of 0.05. Out of the 160 DEGs, 95 genes were upregulated, while 65 genes were downregulated. (Fig. 3G).

### Identification of the Key Module Genes of Sepsis

**Fig. 4:**
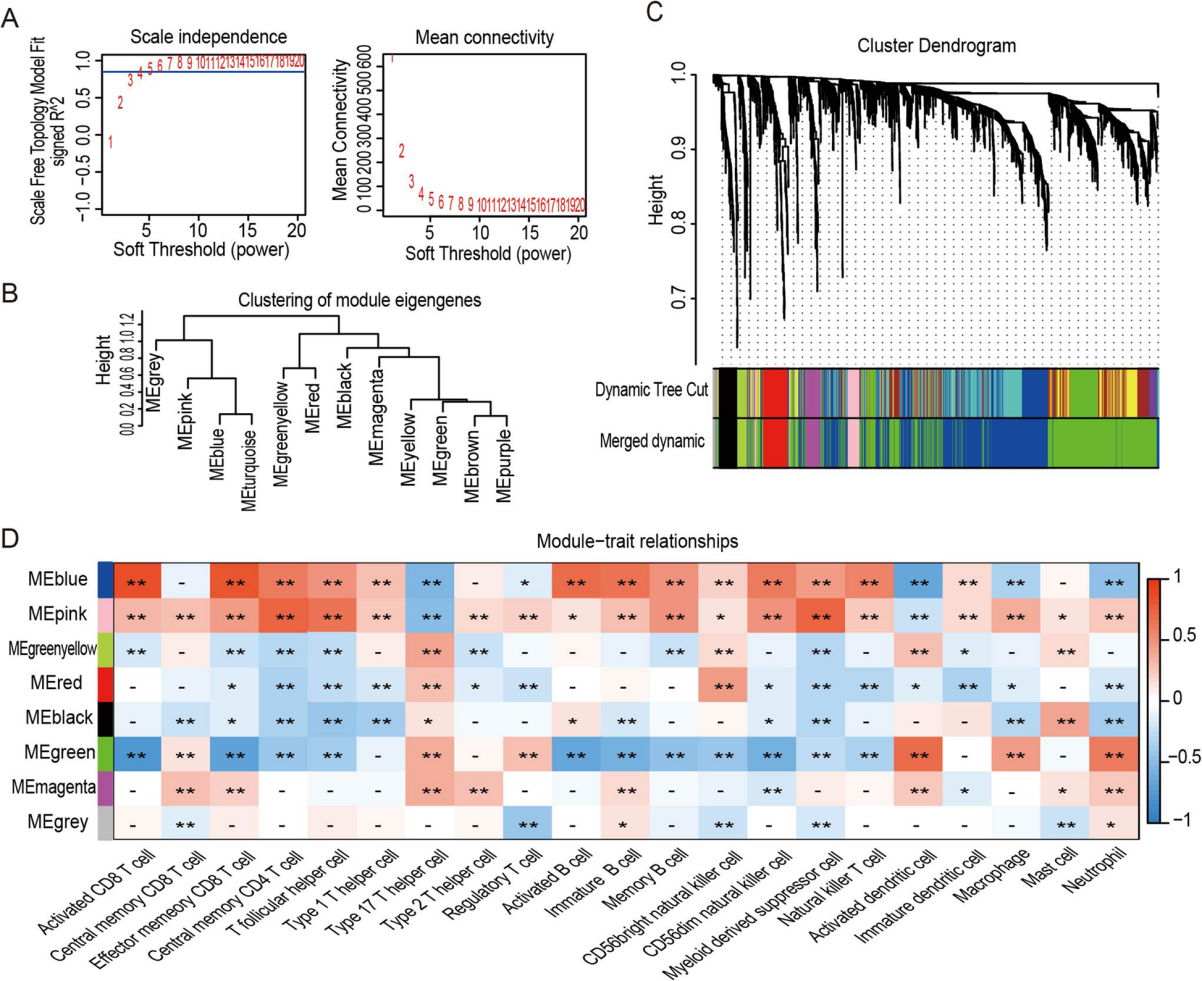
Construction of co-expression modules for sepsis samples. **A** Scale-free index analysis and mean connectivity analysis for selecting the best soft power. **B** Co-expression similarity of all modules based on the hierarchical clustering of module eigengenes. The cut height of 0.3 was chosen to merge similar modules. **C** The cluster dendrogram and color display of co-expression network modules for all genes. The first color row underneath the dendrogram shows the WGCNA module assignment obtained by the dynamic tree cut method. The bottom color row shows the merged modules based on a correlation threshold of 0.7. **D** Heatmap of the correlation between module eigengenes and immune cells. The color spectrum represents the correlation coefficient ranging from -1 to 1. *p < 0.05, **p < 0.01

To determine the co-expression gene modules for the sepsis specimens, WGCNA was carried out. The best soft power for WGCNA was 6 (Fig. 4A). The modules were then grouped based on their correlations, while a "mergeCutHeight" setting of 0.3 was established to combine similar modules (Fig. 4B). Hierarchical clustering was performed to create a dendrogram, with the short vertical line representing a gene and the branches representing co-expressed genes. Altogether, 2484 DEGs were classed into 7 module eigengenes (MEs), including MEblue, MEpink, MEgreenyellow, MEred, MEblack, MEgreen, MEmagenta, and MEgrey (Fig. 4C). The modules that were highly correlated with immune cell infiltration were selected based on the relationship between MEs and immune cells as shown in Fig. 4D. MEblue, MEpink, and MEgrey were significantly associated with immune cell infiltration. Therefore, a total of 2090 genes from these three modules were extracted for subsequent analysis.

### Analysis of Functional Enrichment and the Development of a PPI Network

**Fig. 5:**
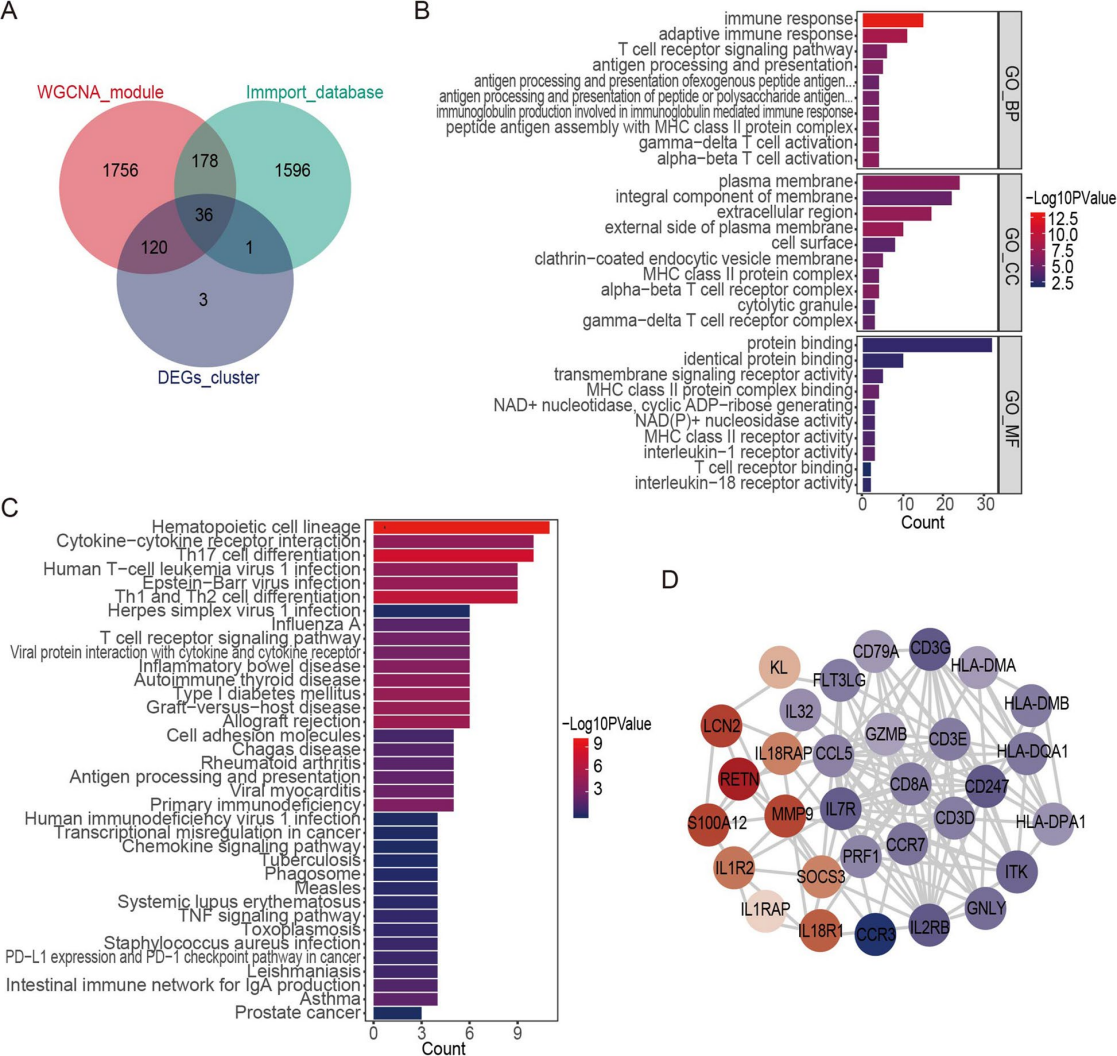
Identification of IRDEGs and enrichment analysis. **A** Venn diagram displaying the identified IRDEGs. **B** Bar chart of IRDEG GO enrichment analysis results. **C** Bar chart of IRDEG KEGG enrichment analysis results. **D** The PPI network of the IRDEGs

To identify the IRDEGs, 1811 IRGs were extracted from the IMMPORT database and then overlapped with 160 DEGs specific to sepsis immune subtypes and 2090 key module genes. As shown in Fig. 5A,36 IRDEGs were identified—Supplementary material 4. To further investigate the biological functions of the IRDEGs, the pathway enrichment analysis was performed. The results of the GO enrichment analysis showed that IRDEGs were mainly related to the adaptive immune response, antigen presentation, and the T cell receptor signaling pathway (Fig. 5B). The KEGG enrichment analysis showed that IRDEGs were mainly associated with cytokine-cytokine receptor interantions, adaptive immune pathways, and cell differentiation (Fig. 5C). The PPI network was constructed for the IRDEGs, consisting of 31 nodes and 142 interaction pairings (Fig. 5D).

### Development and validation of the diagnostic model

**Fig. 6:**
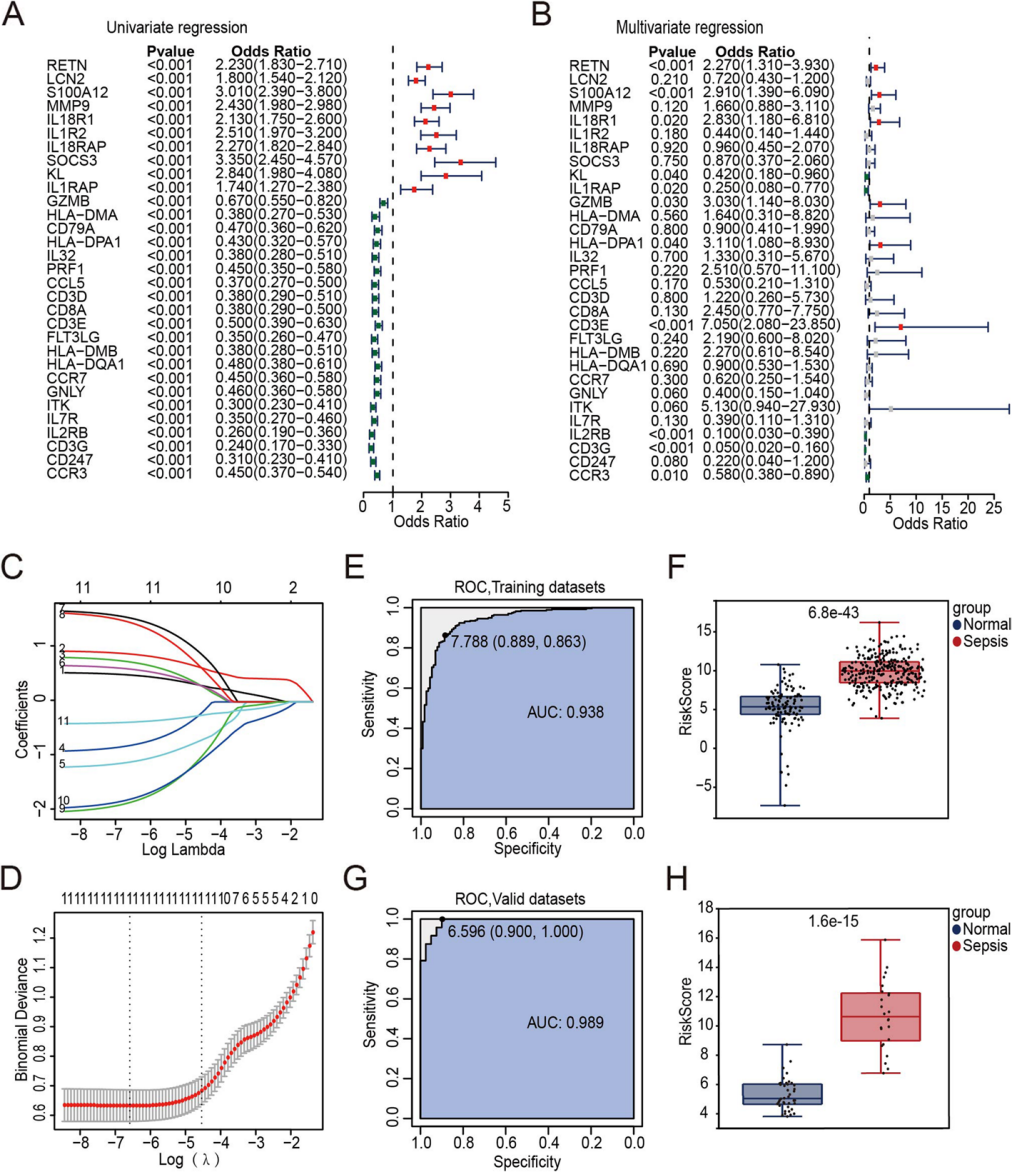
Construction and validation of the diagnostic model. **A** Univariable Cox regression analysis results. **B** Multiple Cox independent prognosis analysis results. **C** LASSO coefficient profiles of the candidate genes. **D** Relationship between partial likelihood deviance and log(λ). **E** ROC curve of the diagnostic model using the training dataset. **F** Boxplots of the risk core distribution using the training dataset. **G** ROC curve of the diagnostic model using the validation dataset. **H** Boxplots of the risk score distribution using the validation dataset

Based on the expression values of the 31 hub genes obtained from the PPI network, with a *p* value of < 0.05 as a filter, univariable Cox regression analysis and multivariable Cox regression analysis were carried out. Subsequently, 11 genes associated with sepsis diagnosis were obtained, including RETN, S100A12, IL18R1, KL, IL1RAP, GZMB, HLA-DPA1, CD3E, IL2RB, CD3G, and CCR3 (Fig. 6A, 6B, *p* < 0.05). Lasso regression analysis was also performed on the 11 genes in order to achieve dimensionality reduction. The regression coefficients of the 11 genes were obtained based on the optimal penalty value lambda (Fig. 6C, 6D). The following regression coefficients were employed to score the diagnostic risk model: Risk Score = 0.485*RETN + 0.864*S100A12 + 0.720*IL18R1 + -0.787*KL + -1.086*IL1RAP + 0.594*GZMB + 1.517*HLA-DPA1 + 1.424*CD3E + -1.820*IL2RB + -1.759*CD3G + -0.367*CCR3.

The predicted risk score classification for the sepsis and the normal control groups was analysed and the obtained AUC value was 0.938 (Fig. 6E). The risk score of sepsis patients was significantly higher than that of the control group (Fig. 6F, *p* < 0.001). To assess the validity of the diagnostic risk score model, we used the risk score to classify the sepsis and normal groups in the GSE154918 dataset. The AUC value in the validation set was 0.989 (Fig. 6G) and the risk score of sepsis patients (*n* = 24) was also higher than that of the control group (*n* = 40) (Fig. 6H, *p* < 0.001). The findings also showed that the diagnosis made according to the independent dataset GSE154918 was with high accuracy. It also confirmed the portability of the sepsis diagnostic model.

### Expression validation and immune correlation analysis of diagnostic genes

**Fig. 7:**
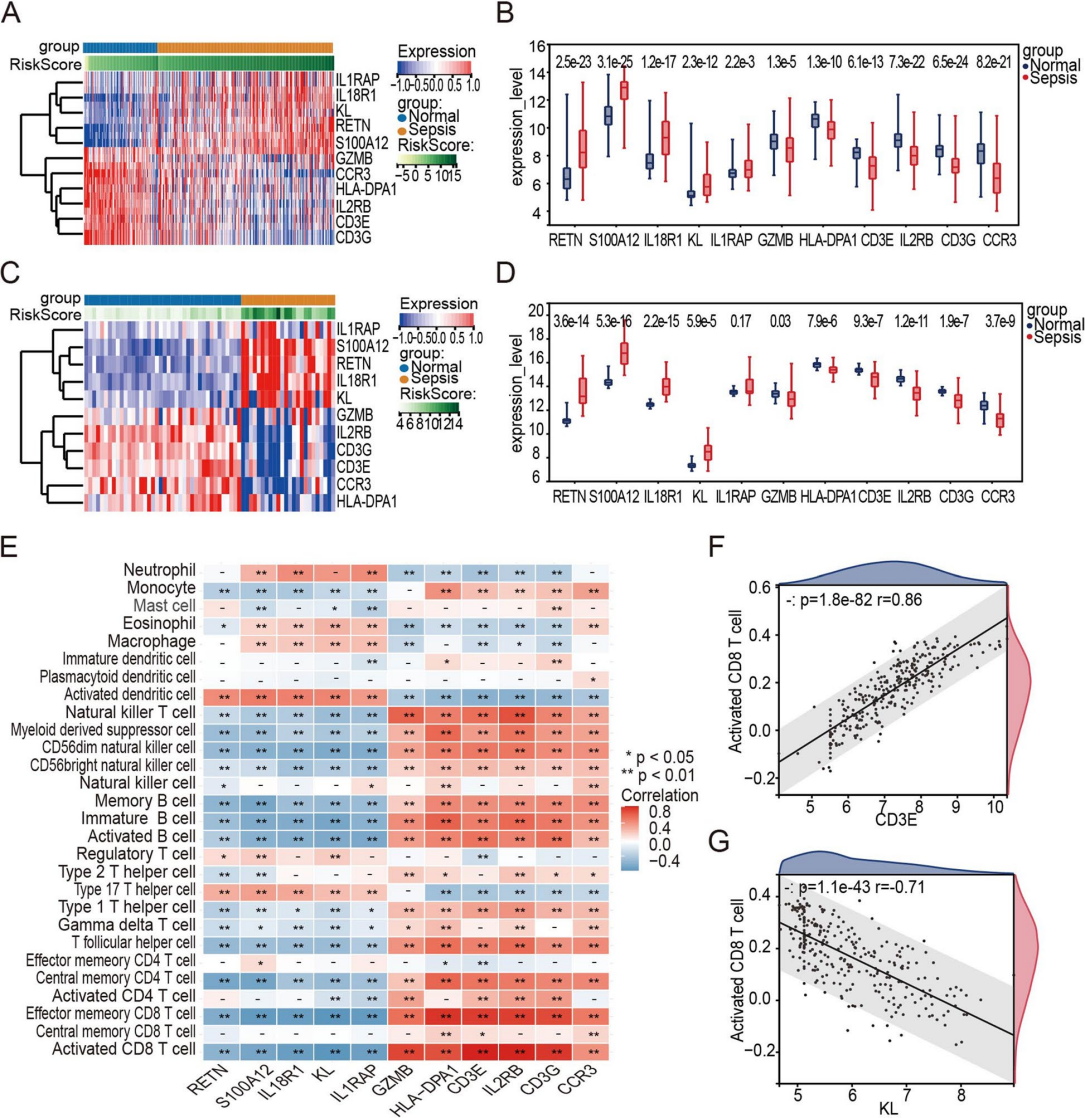
Expression validation and immune correlation analysis of diagnostic genes. **A** Expression heatmap of the 11 diagnostic genes in the training set. **B** Expression box plot of the 11 diagnostic genes in the training set. **C** Expression heatmap of the 11 diagnostic genes in the validation set. **D** Expression box plot of the 11 diagnostic genes in the validation set. **E** The correlation between diagnostic genes and immune cells. *p < 0.05, **p < 0.01, ***p < 0.001. **F** CD3E expression was positively correlated with activated CD8 + T cell infiltration (*p* = 1.18e—82). **G** KL expression was negatively correlated with activated CD8 + T cell infiltration (*p* = 1.1e—43)

The expression values of each diagnostic gene were extracted from the training datasets and the validation dataset respectively. The expression heatmap (Fig. 7A, 7C) and box plot (Fig. 7B, 7D) were drawn combined with the grouping of samples (Sepsis and Normal). As shown in these figures, the expression levels of RETN, S100A12, IL18R1, and KL were higher in the sepsis group, while that of GZMB, HLA-DPA1, CD3E, IL2RB, CD3G, and CCR3 were higher in the normal group (*p* < 0.05). Then, the correlations between diagnostic genes and immune cells were analysed, and the correlation heatmap (Fig. 7E) showed the immune correlation results. Correlation scatter plots of the gene-cell relationship with the largest positive correlation coefficient and the negative correlation coefficient are shown, indicating that CD3E expression was positively and closely related to the level of activated CD8 + T cell infiltration (Fig. 7F, *p* = 1.18e—82), while KL expression was negatively and closely related to the level of activated CD8 + T cell infiltration (Fig. 7G, *p* = 1.1e—43).

## Discussion

Despite advancements in diagnostic techniques and treatments, the prognosis of sepsis patients remains unfavourable [22]. Sepsis is a complex condition influenced by a multifaceted immunological network, involving various signalling molecules, transcription factors, and metabolic reprogramming [23, 24]. Therefore, investigating the interplay between immune cells and sepsis and identifying immune-related diagnostic genes is crucial to improve the accuracy of sepsis diagnosis and treatment efficacy.

Sepsis is a complex disease process that is characterized by heterogeneous and dynamic manifestations [25]. A single time point study may not fully capture the key changes that occur during the progression of sepsis. Therefore, we believe that determining the differentially expressed genes at expression between different time points in sepsis can provide a more comprehensive understanding of the pathological processes involved. In this study, we chose to include samples from multiple time points rather than the earliest time point or a single time point, aiming to capture the temporal dynamics of gene expression in sepsis. This approach allowed us to identify genes that exhibited significant changes and understand their potential roles in different stages of sepsis. Additionally, studying the complete spectrum of sepsis allowed us to investigate the progression and pathophysiology of the disease more comprehensively. On the other hand, we acknowledge that there is indeed a risk of duplicate counting when including samples from multiple time points in the analysis. If samples from the same individual are collected at different time points, these samples may have similar gene expression patterns, introducing the issue of duplication. However, we believe it is precisely this repetition that helps identify which genes are most representative and stable during the early stages of sepsis.

In this study, we intergrated 4 GEO datasets to comprehensively investigate the expression landscape of sepsis in an unbiased manner. Based on this integration, there were differences in the abundance of 22 immune cells between the sepsis and control groups, among which macrophages, neutrophils, mast cells, Th17 cells, and Treg cells showed significantly higher abundance in the sepsis group compared to the control group. We also performed an unsupervised consensus clustering analysis of differential immune cell profiles from sepsis samples, thereby demonstrating that there were two robust sepsis subtypes. We noticed that one of the subtypes (subtype 2) identified a group of patients with features of relatively active cellular metabolism and biosynthesis associated with a better prognosis.

In our study, the objective was to identify DEGs as comprehensively as possible to explore key immune-related mechanisms involved in sepsis. Therefore, we selected a threshold of |LogFC|< 0.263 for filtering DEGs instead of using 1 as the threshold when comparing between the sepsis samples and normal samples. We were aware that setting the threshold to 1 would provide higher filtering precision by only selecting genes with larger fold changes. However, in our preliminary analyses, we found that setting the threshold to 1 resulted in a significantly low number of DEGs, and we could be potentially missing some significant genes. To ensure that our filtering of DEGs was not overly stringent, we conducted multiple experiments and trials, ultimately selecting 0.263 as the threshold.

Cellular metabolism is the basis of cellular activity. During glycolysis, sufficient biomolecules and energy are produced to support the biological development, differentiation, and proliferation of immune cells [26, 27]. Nonetheless, it is important to note that a high rate of glycolysis can result in lactate buildup and immunosuppression [28, 29]. Metabolic disorders are largely responsible for the immune imbalance observed during sepsis [30]. In recent years, new concepts for classifying chemicals produced during metabolic overload based on metabolism-associated molecular patterns (MAMPs) have been described [31]. As MAMPs play a vital role in the pathophysiology and the progression of sepsis, perhaps targeting them could provide attractive strategies for treating sepsis [32].

Neutrophils and macrophages are important components of the innate immune system, and their roles in sepsis have been extensively documented [33–35]. Although neutrophils are essential for preventing infection under normal circumstances, their biological function is compromised in sepsis patients, which contributes to the dysregulation of the immune responses [36]. Our findings align with those of previous research that suggest a strong correlation between sepsis and an increase in circulating neutrophil numbers [37]. Surprisingly, during the clustering analysis of sepsis patients in our study, the group of patients with features of relatively low abundance of neutrophils (subtype 1) was associated with a worse prognosis. This may be attributed to an immunosuppressed phenotype, the presence of abnormal neutrophils, or immature neutrophils in these patients [38, 39].

Macrophages play a crucial role in orchestrating the immune response to sepsis, as they serve as the body's initial line of defence. These cells can exhibit different phenotypes, characterized as M1- or M2-like, with distinct functions in response to modifications of the tissue microenvironment [40]. Despite their importance, there are still many limitations in research focusing on the targeted regulation of macrophage polarization in sepsis [41]. Our study found that there was a greater survival rate in the subtype 2 group, and the patients in this group had a large amount of M1 macrophage polarization. This finding suggests that targeted regulation that increases M1-like macrophage polarization or decreases M2-like macrophage polarization could offer new therapeutic possibilities for sepsis management. These insights pave the way for potential interventions to modulate macrophage phenotypes and improve sepsis outcomes.

Mast cells play a critical role in combatting pathogens since they serve as key immune effectors and modulatory cells in the human body that aid innate and adaptive immunity [42]. Nonetheless, research into the role of MCs in sepsis is extremely limited. Although a few existing studies suggest that mast cells may strengthen the host's resistance to infection [43, 44], contradictory findings have also emerged, indicating that mast cells could contribute to dysregulated host responses, potentially leading to increased morbidity and mortality [45, 46]. Consequently, further research employing proteomic or genomic methods is necessary to comprehensively elucidate the impact of MCs on sepsis [47, 48]. These investigations would provide crucial insights into the precise role and mechanisms by which MCs influence sepsis development and progression.

T cells play an indispensable role in adaptive immunity and are crucial in the immunosuppressive state that accompanies sepsis [49]. Th17 cells are a subpopulation of T helper cells that have been linked to autoimmune conditions and are identified based on their production of IL-17 [50]. Notably, Th17 cells can protect the body against extracellular infections that colonize mucosal surfaces [51]. Consistent with these observations, our findings indicate an increased level of Th17 cells in sepsis patients' blood samples that were collected upon admission, substantiating the findings of previous studies [52]. On the other hand, Tregs are important immunoregulatory cells with the capacity to inhibit the proinflammatory impacts of effector T cells [53]. Additionally, Tregs have been linked to higher immune paralysis and mortality in sepsis patients, according to previous reports [54]. Thus, targeting Treg immunometabolism could provide new therapeutic options for treating sepsis [55]. By targeting Treg immunometabolism, it may be possible to modulate immune responses and improve the outcomes of patients with sepsis.

A biological function analysis was also performed on the 36 IRDEGs that were identified in our work and the findings indicated that they were predominantly engaged in the adaptive immune response, antigen presentation, and interactions between cytokine and cytokine receptors. T cells, B cells, and dendritic cells are just a few of the cell types involved in the adaptive immune response, and these cells play a critical role in reducing inflammation and tissue damage following infection and restoring general host immunological homeostasis [56]. The antigen-presenting process serves as a crucial link between innate and adaptive immunity, facilitating the recognition of antigenic epitopes by T cells on the surface of antigen-presenting cells (APCs), which include dendritic cells, monocytes, and macrophages [57]. This process is vital for the initiation and regulation of specific immune responses. By presenting antigens to T cells, APCs contribute to the activation of adaptive immune cells and the subsequent elimination of pathogens. Understanding the intricate mechanisms underlying antigen presentation is essential for unravelling the dynamic interplay between innate and adaptive immunity in response to infection.

A diagnostic model incorporating 11 immune-related genes was developed and demonstrated strong predictive performance for sepsis detection. Our findings showed that the expression of RETN, S100A12, IL18R1, and KL was higher in the sepsis group, while the expression of GZMB, HLA-DPA1, CD3E, IL2RB, CD3G, and CCR3 was higher in the control group. Among these genes, CD3E expression was the most positively correlated with activated CD8 T cell infiltration, while Klotho (KL) expression was the most negatively correlated with activated CD8 T cell infiltration. CD3E is a component of the TCR-CD3 complex that exists on the T-lymphocyte cell surface and is critical in the adaptive immune response [58]. It has been reported that patients with organ dysfunction showed lower expression of CD3E [59]. Previous work also showed that CD3E expression was downregulated in the sepsis group, which is consistent with the findings of our study [60]. Klotho encodes a type-I membrane protein that is related to beta-glucosidases [61]. It is interesting to note that the lipopolysaccharide (LPS) injection-induced sepsis model showed decreased KL mRNA expression [62]. This finding is inconsistent with our results; however, this difference can be partially explained because of the different sample sources, although validation will require further study.

There is also limitations to this study. For example, we provided validation only in historical independent datasets, not in a prospective cohort. Furthermore, as the datasets incorporated did not provide a comprehensive description of the clinical characteristics of patients, such as the presence of coexisting diseases, specific sites of infection, and relevant laboratory findings, we are unable to ascertain the specific ability of these diagnostic genes in identifying the pertinent features associated with sepsis. We recognize that further work is needed to further validate our findings.

In conclusion, in this study, we provided a comprehensive insight into the immune features associated with sepsis and successfully identified a robust diagnostic model based on 11 diagnostic genes that can be used for sepsis detection. The findings of this research contribute to the advancement of precision medicine approaches for sepsis management and can facilitate the development of novel targeted therapies. These results signify a significant step towards improving the diagnosis and treatment of sepsis, with the goal of ultimately improving patient outcomes and reducing the burden of this life-threatening condition. Further studies and clinical validations are warranted to translate these findings into clinical practice and to fully understand the therapeutic potential of the identified genes and diagnostic model in sepsis management.

### Supplementary Information

**Additional file 1. **

**Additional file 2. **

**Additional file 3. **

**Additional file 4. **

## Data Availability

The datasets presented in this study can be download from GEO database (https://www.ncbi.nlm.nih.gov/geo/). GEO accession ID: GSE28750: https://www.ncbi.nlm.nih.gov/geo/query/acc.cgi?acc=GSE28750 GSE54514: https://www.ncbi.nlm.nih.gov/geo/query/acc.cgi?acc=GSE54514 GSE69063: https://www.ncbi.nlm.nih.gov/geo/query/acc.cgi?acc=GSE69063 GSE69528: https://www.ncbi.nlm.nih.gov/geo/query/acc.cgi?acc=GSE69528 GSE154918: https://www.ncbi.nlm.nih.gov/geo/query/acc.cgi?acc=GSE154918

## Abbreviations

APC: Antigen-presenting cells
AUC: Area under the ROC curve
BP: Biological process
CC: Cellular component
CDF: Cumulative distribution function
CIBERSORT: Cell-type identification by estimating relative subsets of RNA transcripts
DCs: Dendritic cells
FC: Fold change
GEO: Gene Expression Omnibus
GO: Gene ontology
IFN-γ: Interferon-γ
IL-1β: Interleukin-1β
IMMPORT: Immunology Database and Analysis Portal
IRDEGs: Immune-related Differential Genes
IRGs: Immune-related genes
KEGG: Kyoto Encyclopedia of Genes and Genomes
LASSO: Least absolute shrinkage and selection operator
MAMPs: Metabolism-associated molecular patterns
MEs: Module eigengenes
MF: Molecular function
MODS: Multiple organ dysfunction syndromes
NES: Normalized enrichment score
PPI: Protein-Protein Interaction Network
ROC: Receiver operating characteristic
ssGSEA: Single-sample gene set enrichment analysis
TNF-α: Tumour necrosis factor
TOM: Topological overlap matrix
WCGNA: Weighted gene co-expression network analysis

## References

[1] Shankar-Hari M, Phillips GS, Levy ML, Seymour CW, Liu VX, Deutschman CS (2016). Developing a New Definition and Assessing New Clinical Criteria for Septic Shock: For the Third International Consensus Definitions for Sepsis and Septic Shock (Sepsis-3). JAMA.

[2] Rudd KE, Johnson SC, Agesa KM, Shackelford KA, Tsoi D, Kievlan DR (2020). Global, regional, and national sepsis incidence and mortality, 1990–2017: analysis for the Global Burden of Disease Study. The Lancet.

[3] Peltan ID, Mitchell KH, Rudd KE, Mann BA, Carlbom DJ, Hough CL (2017). Physician Variation in Time to Antimicrobial Treatment for Septic Patients Presenting to the Emergency Department. Crit Care Med.

[4] Leligdowicz A, Matthay MA (2019). Heterogeneity in sepsis: new biological evidence with clinical applications. Crit Care.

[5] Barichello T, Generoso JS, Singer M, Dal-Pizzol F (2022). Biomarkers for sepsis: more than just fever and leukocytosis—a narrative review. Crit Care.

[6] Lazzaro A, De Girolamo G, Filippi V, Innocenti GAO, Santinelli LAOX, Ceccarelli GAO (2022). The Interplay between Host Defense, Infection, and Clinical Status in Septic Patients: A Narrative Review. Int J Mol Sci..

[7] Jarczak DAO, Nierhaus AAO (2022). Cytokine Storm-Definition, Causes, and Implications. Int J Mol Sci..

[8] Torres LK, Pickkers P, van der Poll T (2022). Sepsis-Induced Immunosuppression. Annu Rev Physiol.

[9] Barrett T, Suzek TO, Troup DB, Wilhite SE, Ngau WC, Ledoux P (2005). NCBI GEO: mining millions of expression profiles–database and tools. Nucleic acids research..

[10] Leek JT, Johnson WE, Parker HS, Jaffe AE, Storey JD (2012). The sva package for removing batch effects and other unwanted variation in high-throughput experiments. Bioinformatics (Oxford, England).

[11] Hänzelmann S, Castelo R, Guinney J (2013). GSVA: gene set variation analysis for microarray and RNA-Seq data. BMC Bioinformatics.

[12] Chen B, Khodadoust MS, Liu CL, Newman AM, Alizadeh AA (2018). Profiling Tumor Infiltrating Immune Cells with CIBERSORT. Methods in molecular biology (Clifton, NJ).

[13] Wilkerson MD, Hayes DN (2010). ConsensusClusterPlus: a class discovery tool with confidence assessments and item tracking. Bioinformatics (Oxford, England).

[14] Smyth GK, Gentleman R, Carey VJ, Huber W, Irizarry RA, Dudoit S (2005). limma: Linear Models for Microarray Data. Bioinformatics and Computational Biology Solutions Using R and Bioconductor.

[15] Langfelder P, Horvath S (2008). WGCNA: an R package for weighted correlation network analysis. BMC Bioinformatics.

[16] Sherman BT, Hao M, Qiu J, Jiao X, Baseler MW, Lane HC (2022). DAVID: a web server for functional enrichment analysis and functional annotation of gene lists (2021 update). Nucleic Acids Res.

[17] Kanehisa M, Goto S (2000). KEGG: kyoto encyclopedia of genes and genomes. Nucleic Acids Res.

[18] Ashburner M, Ball CA, Blake JA, Botstein D, Butler H, Cherry JM (2000). Gene ontology: tool for the unification of biology. The Gene Ontology Consortium Nature genetics.

[19] Kanehisa M (2019). Toward understanding the origin and evolution of cellular organisms. Protein Sci.

[20] Kanehisa M, Furumichi M, Sato Y, Kawashima M, Ishiguro-Watanabe M (2023). KEGG for taxonomy-based analysis of pathways and genomes. Nucleic Acids Res.

[21] Szklarczyk D, Morris JH, Cook H, Kuhn M, Wyder S, Simonovic M (2017). The STRING database in 2017: quality-controlled protein-protein association networks, made broadly accessible. Nucleic Acids Res.

[22] Schmidt K, Gensichen J, Fleischmann-Struzek C, Bahr V, Pausch C, Sakr Y (2020). Long-Term Survival Following Sepsis. Deutsches Arzteblatt international.

[23] Fitzpatrick SF. Immunometabolism and Sepsis: A Role for HIF? Front Mol Biosci. 2019;6:85. 10.3389/fmolb.2019.00085 PMC6742688 31555665

[24] Zhang Y-y, Ning B-t (2021). Signaling pathways and intervention therapies in sepsis. Signal Transduct Target Ther.

[25] van der Poll T, Shankar-Hari M, Wiersinga WJ (2021). The immunology of sepsis. Immunity.

[26] Gauthier T, Chen WJFiI. Modulation of macrophage immunometabolism: A new approach to fight infections. 2022;13:780839. 10.3389/fimmu.2022.780839 PMC8825490 35154105

[27] Krawczyk CM, Holowka T, Sun J, Blagih J, Amiel E, DeBerardinis RJ (2010). Toll-like receptor–induced changes in glycolytic metabolism regulate dendritic cell activation. Blood..

[28] Morioka S, Perry JS, Raymond MH, Medina CB, Zhu Y, Zhao L (2018). Efferocytosis induces a novel SLC program to promote glucose uptake and lactate release.

[29] Puig-Kroeger A, Pello O, Selgas R, Criado G, Bajo M, Sanchez-Tomero JA (2003). Peritoneal dialysis solutions inhibit the differentiation and maturation of human monocyte-derived dendritic cells: effect of lactate and glucose-degradation products.

[30] Liu J, Zhou G, Wang X, Liu D (2022). Metabolic reprogramming consequences of sepsis: adaptations and contradictions. Cell Mol Life Sci.

[31] Wang X, Wang Y, Antony V, Sun H, Liang G (2020). Metabolism-Associated Molecular Patterns (MAMPs). Trends Endocrinol Metab.

[32] Zhu XX, Zhang WW, Wu CH, Wang SS, Smith FG, Jin SW (2022). The Novel Role of Metabolism-Associated Molecular Patterns in Sepsis. Front Cell Infect Microbiol.

[33] Stearns-Kurosawa DJ, Osuchowski MF, Valentine C, Kurosawa S, Remick DG (2011). The Pathogenesis of Sepsis. Annu Rev Pathol.

[34] Brown KA, Brain SD, Pearson JD, Edgeworth JD, Lewis SM, Treacher DF (2006). Neutrophils in development of multiple organ failure in sepsis. Lancet (London, England).

[35] Cavaillon J-M (2005). Adib-Conquy MJCcm. Monocytes/macrophages and sepsis.

[36] Shen XF, Cao K, Jiang JP, Guan WX, Du JF (2017). Neutrophil dysregulation during sepsis: an overview and update. J Cell Mol Med.

[37] Agnello L, Giglio RV, Bivona G, Scazzone C, Gambino CM, Iacona A (2021). The Value of a Complete Blood Count (CBC) for Sepsis Diagnosis and Prognosis. Diagnostics (Basel, Switzerland)..

[38] Demaret J, Venet F, Friggeri A, Cazalis MA, Plassais J, Jallades L (2015). Marked alterations of neutrophil functions during sepsis-induced immunosuppression.

[39] Wang J-F, Li J-B, Zhao Y-J, Yi W-J, Bian J-J, Wan X-J (2015). Up-regulation of programmed cell death 1 ligand 1 on neutrophils may be involved in sepsis-induced immunosuppression: an animal study and a prospective case-control study. Anesthesiology..

[40] Liu Y-C, Zou X-B, Chai Y-F, Yao Y-M (2014). Macrophage Polarization in Inflammatory Diseases. Int J Biol Sci.

[41] Chen X, Liu Y, Gao Y, Shou S, Chai Y (2021). The roles of macrophage polarization in the host immune response to sepsis. Int Immunopharmacol.

[42] Urb M, Sheppard DC (2012). The Role of Mast Cells in the Defence against Pathogens. PLoS Pathog.

[43] Sutherland RE, Olsen JS, McKinstry A, Villalta SA, Wolters PJ (2008). Mast cell IL-6 improves survival from Klebsiella pneumonia and sepsis by enhancing neutrophil killing. J Immunol..

[44] Thakurdas SM, Melicoff E, Sansores-Garcia L, Moreira DC, Petrova Y, Stevens RL (2007). The mast cell-restricted tryptase mMCP-6 has a critical immunoprotective role in bacterial infections. J Biol Chem..

[45] Piliponsky AM, Chen C-C, Grimbaldeston MA, Burns-Guydish SM, Hardy J, Kalesnikoff J (2010). Mast cell-derived TNF can exacerbate mortality during severe bacterial infections in C57BL/6-KitW-sh/W-sh mice. Am J Pathol..

[46] Dahdah A, Gautier G, Attout T, Fiore F, Lebourdais E, Msallam R (2014). Mast cells aggravate sepsis by inhibiting peritoneal macrophage phagocytosis.

[47] Piliponsky AM, Acharya M, Shubin NJ. Mast Cells in Viral, Bacterial, and Fungal Infection Immunity. Int J Mol Sci. 2019;20(12):2851. 10.3390/ijms20122851 PMC6627964 31212724

[48] Johnzon C-F, Rönnberg E, Pejler G (2016). The Role of Mast Cells in Bacterial Infection. Am J Pathol.

[49] Boomer JS, To K, Chang KC, Takasu O, Osborne DF, Walton AH (2011). Immunosuppression in patients who die of sepsis and multiple organ failure.

[50] Maddur MS, Miossec P, Kaveri SV, Bayry J (2012). Th17 Cells: Biology, Pathogenesis of Autoimmune and Inflammatory Diseases, and Therapeutic Strategies. Am J Pathol.

[51] Peck A, Mellins ED (2010). Precarious balance: Th17 cells in host defense. Infect Immun.

[52] Brunialti MK, Santos MC, Rigato O, Machado FR, Silva E, Salomao R. Increased percentages of t helper cells producing il-17 and monocytes expressing markers of alternative activation in patients with sepsis. PLoS ONE. 2012;7(5):e37393. 10.1371/journal.pone.0037393 PMC3365066 22693573

[53] Arce-Sillas A, Álvarez-Luquín DD, Tamaya-Domínguez B, Gomez-Fuentes S, Trejo-García A, Melo-Salas M, et al. Regulatory T cells: molecular actions on effector cells in immune regulation. 2016;2016:1720827. 10.1155/2016/1720827 PMC4889823 27298831

[54] Monneret G, Debard AL, Venet F, Bohe J, Hequet O, Bienvenu J (2003). Marked elevation of human circulating CD4+CD25+ regulatory T cells in sepsis-induced immunoparalysis. Crit Care Med.

[55] Kumar V (2018). T cells and their immunometabolism: A novel way to understanding sepsis immunopathogenesis and future therapeutics. Eur J Cell Biol.

[56] Brady J, Horie S, Laffey JG (2020). Role of the adaptive immune response in sepsis. Intensive Care Med Exp.

[57] Gaudino SJ, Kumar P (2019). Cross-Talk Between Antigen Presenting Cells and T Cells Impacts Intestinal Homeostasis, Bacterial Infections, and Tumorigenesis. Front Immunol.

[58] Barber EK, Dasgupta JD, Schlossman SF, Trevillyan JM, Rudd CE (1989). The CD4 and CD8 antigens are coupled to a protein-tyrosine kinase (p56lck) that phosphorylates the CD3 complex. Proc Natl Acad Sci U S A..

[59] Menéndez R, Méndez R, Almansa R, Ortega A, Alonso R, Suescun M, et al. Simultaneous Depression of Immunological Synapse and Endothelial Injury is Associated with Organ Dysfunction in Community-Acquired Pneumonia. J Clin Med. 2019;8(9):1404. 10.3390/jcm8091404 PMC6780106 31500177

[60] Lu J, Li Q, Wu Z, Zhong Z, Ji P, Li H (2020). Two gene set variation indexes as potential diagnostic tool for sepsis. American journal of translational research.

[61] Klotho K-O M (2010). Pflugers Archiv European. J Physiol.

[62] Ohyama Y, Kurabayashi M, Masuda H, Nakamura T, Aihara Y, Kaname T (1998). Molecular cloning of rat klotho cDNA: Markedly decreased expression of klotho by acute inflammatory stress. Biochem Biophys Res Commun.

